# The Surgical Treatment of Cancer

**Published:** 1905-09

**Authors:** 


### *SOCIETY REPORTS.*

---

### THE SURGICAL TREATMENT OF CANCER.*

Dr. M. B. Hutchins opened the discussion. The element of hope enters greatly into the surgical treatment of this disease, many cases being operated upon when too late, only because the patient hopes for cure, but the doctor shares this feeling, hoping the case may be cured. He believed that very few operators were influenced in their work by motives of gain or love of operating, but that they really desired to give the patient a chance. The result of surgical intervention depends upon the extent of the disease and the metastasis. Early operation is not always effective, as even a recent case may have passed the bounds of successful removal. Often a long-standing case may show favorable limitations. As a rule, however, cases coming early are small, circumscribed and curable. Success depends simply upon our ability to remove all the disease in one piece, the avoidance of reinoculation during the dissection and forcing particles of disease beyond the operated area by excessive manipulation.

In the surgical removal of cancer one must have plenty of room to leave a wide margin of apparently healthy tissue, hence regions such as of the neck are favorable, while the nose, ear or naso-canthal areas are unfavorable.

Dr. Hutchins stated that he had operated upon practically every region, save the cavities of the trunk, for cancer, from the thigh to the scalp. On the scalp a wide excision is possible, but its depth is limited by the skull, and closure of the wound is difficult. Operations upon the external ear are disfiguring, on the ear and adjacent tissues circumscribed. On the nose or naso-canthal region wide dissection is impracticable. Cancer involving the eye impossible of clean removal. Cancer of the cheek, lip or neck are susceptible of wide dissection with plastic repair. While often the submental and submaxillary glands become involved in lower-

*Discussion of Meeting of Fulton County Medical Society, Atlanta, August 17, 1905.

lip cases, routine removal of the glands with the lip is scarcely justifiable unless they are palpably diseased.

It was the speaker's observation that cancer above the line of the mouth (being of a different type) showed glandular metastasis much more rarely than where the disease was below the line. Most cases of cancer of the tongue are not helped by operation, having become too extensive and shown early metastasis.

All cases of cancer of the breast show a varying time limit of relief or cure of not over twenty-five per cent, regardless of thoroughness and extent of removal. There is no doubt that many so-called recurrences after a period of years or months are not recurrences, but new developments, as in the other breast or in the cicatricially distorted tissues—while the other outlying recurrent skin nodes are doubtless metastatic recurrences due to manipulation extension. These often occur after the widest possible Halsted dissection.

Wilby Meyer is probably correct when he says that extension to glands above the clavicle always extinguishes hope of cure.

Cancer of the uterus rarely is cured by excision, it being impossible to remove all diseased tissues.

With stomach and intestinal cases the prognosis is almost totally hopeless, owing to the difficulty of early diagnosis and the anatomy of these regions.

Mayo Robson mentions remarkable cases—there have been a few even in this State—of recovery from malignant internal disease after a simple exploratory operation, some nerve impulse or shock or some antitoxic and curative element being developed. Dr. Hutchins mentioned a case recently at a sanatorium in Atlanta, exploratory operation on a young man, osteo-sarcoma of the fossa of the right ilium, closure of the wound, intense secondary infection, pus as from an appendical abscess, burrowing even along coupart's ligament; man apparently moribund day after day, carried home hopeless and at last accounts "going to baseball games." The speaker did not hesitate to say the surgical was the best treatment we have provided, if the disease was so situated as to permit of proper excision and the patient's consent to operation could be obtained.

*Dr. Thrash:* I would like to say a word on this subject from the standpoint of one who has done general practice for a number of years. All primary malignant growths, in the early stage of their existence, are benign, and the antiquated advice so often even yet given patients, to never disturb abnormal growths till they disturb you, has given many an innocent growth time to reach a degree of malignancy that no skill, surgical or otherwise, can repair the damage that delay has caused.

I go right after every mole, papilloma neous, or any variety of incipient tumor, wherever I find it, if the patient will allow such procedure. Whenever a patient calls upon me professionally, and I see upon his body any of these abnormalties, I do not wait for him to ask my advice about them, but I tell him emphatically to get rid of them, and if he has no more confidence in me than to think I am talking for a fee, I at least get him interested and anxious enough for him to casually ask some other doctor about them, and if we would all adopt this plan we would very greatly lessen the mortality from cancers. I have put many a person on the table and nicked out from one to half dozen moles, etc., that he had been carrying since birth, and had looked upon them as of no consequence. This method is decidedly the best means we have at hand in solving the cancer problem, and it is too important to ignore, and too simple not to be practiced by us all.

*Dr Simpson:* How do you get rid of them, Doctor?

*Dr. T.:* I cut them out almost invariably.

*Dr. Cartledge:* I was not quite expecting Dr. Hutchins to give to surgery the chiefest place in the cure of epithelioma. Some of us have been using the X-ray so effectually that we thought we could not do so well by operation.

I have had only six cases, but a perfect result each time. They were all small but quite a positive condition of epithelioma.

In five of these cases operation might have cured, but would have left conspicuous scars, and I have seen operations usually fail in cases more advanced than mine were. They were: one of the lip, three of the cheek, one of the nose and one of the cervix uteri.

Dr. Brawner saw my first case, of the cheek, and told me he was quite sure I could cure it with X-ray from his experience with such cases.

The nose case came to the city expecting operation. He came to me saying the surgeons he had seen told him the X-ray was preferable.

The lip case was sent to me by Dr. Born, of McRae, Ga. Dr. Born was one of the first of us in Georgia to do X-ray work, and after a good experience he thought the ray his best cure, and the patient was more convenient to me than to Dr. Born.

It is important that one should be thoroughly acquainted with his tube and machine and give full length of treatment from the start. Two cases of lupus resisted treatment. I purposely burnt them to the first and then second degree. I cured one by operation. Other returned after operation. I wonder if the violet ray would cure this case. The violet ray is much inferior to X-ray in their usual applications, I'm told.

Dr Hutchins, closing the discussion, said that he purposely omitted all other forms of treatment because the subject was solely surgical. He had probably had as much experience with the ray, being the first man in the South to use it, as anybody. There were cases suitable only for ray treatment and he expected to continue to use it and to have to use it where operation could not be done, or where the patient refused the knife.

He commended Dr. Cartledge's idea of giving these cases full and powerful treatment with rays from the first, no teasing along without sufficient demonstrative force. He endorsed Dr. Thrash's remarks in full, but even as Keene, whose article was referred to, has said, a person with a large number of moles, even though he be a doctor, is not going to allow their universal removal.

Dr. Hutchins especially appreciated Dr. McRae's remarks and their concurrence with his own views and reiterated his belief in the more general usefulness and success of the surgical treatment. He hoped, however, that the future might bring forth a serum, antitoxin or some other remedy which would enable us to gain mastery over the disease.

